# The weight of pills: medication use and fall incidence in Saudi community-dwelling adults aged 50 years and older: a 10-month longitudinal study

**DOI:** 10.3389/fpubh.2026.1889811

**Published:** 2026-09-29

**Authors:** Aqeel M. Alenazi, Norah A. Alhwoaimel, Bader A. Alqahtani

**Affiliations:** 1 Department of Health and Rehabilitation Sciences, College of Applied Medical Sciences in Al-Kharj, Prince Sattam Bin Abdulaziz University, Al-Kharj, Saudi Arabia; 2 King Salman Center for Disability Research, Riyadh, Saudi Arabia

**Keywords:** fall risk, independent living, longitudinal, medication, older adults, polypharmacy, Saudi

## Abstract

**Background:**

Despite the growing body of global evidence on the use of medications associated with a risk of falls, there is a lack of research on medications and future fall incidence in the Saudi population.

**Objective:**

This study aimed to explore the association of the baseline number of medications and polypharmacy with fall incidence at 4–6 and 9-10 months of follow-up among Saudi adults aged 50 years and older.

**Methods:**

A prospective longitudinal study was conducted among 211 Saudi adults aged 50 years and older. Participants were recruited from community settings including hospitals, clinics, malls, and mosques. Data were collected at three assessment time points: baseline (February–April 2022), 4–6 months (August-September 2022), and 9–10 months (November 2022–January 2023). The number of medications and polypharmacy (≥4 medications) were verified during participant interviews at baseline. Fall events (fall vs. no fall) were documented at three time points across 10 months of follow-up, including baseline fall history, fall incidence at 4–6 months, and fall incidence at 9-10 months of follow-up. Cox proportional hazards regression was used to estimate hazard ratios (HRs) and 95% confidence intervals (CIs).

**Results:**

Baseline polypharmacy was significantly associated with an approximately fourfold increase in fall incidence (HR: 3.99, 95% CI: 1.63–9.81; *p* = 0.003) in the final parsimonious model retaining age and the Insomnia Severity Index (ISI) score. The number of medications was not significantly associated with fall incidence in the final adjusted model.

**Conclusion:**

This exploratory study suggests a possible association between baseline polypharmacy and fall incidence over a follow-up period of up to 10 months among community-dwelling Saudi adults aged 50 years and older. However, these findings were based on a small polypharmacy subgroup (*n* = 14) and include only six events, so they should be interpreted with caution. These findings may support further investigation of medication and polypharmacy screening as part of broader fall-risk management strategies for community-dwelling older adults. However, further research is needed to confirm this association and clarify its clinical implications.

## 1 Introduction

The risk of falling is a common public health concern, leading to a significant health burden, functional decline, morbidity, mortality, and increased healthcare costs (1, 2). These fall-related challenges are substantial among older adults, especially among rapidly aging populations in Saudi Arabia. The risk of falls might be linked to various factors, including physical conditions and chronic diseases such as multimorbidity, diabetes, arthritis, hypertension, stroke, cardiovascular diseases, and lower physical activity levels at a relatively younger age in Saudi Arabia (3–10). The prevalence of chronic diseases has increased in Saudi adults aged 50 years and older (11). The rapid rise in the prevalence of chronic conditions is associated with an increase in medication use. This increase in the number of medications may lead to an increased risk of falls due to side effects or interactions, including dizziness, orthostatic hypotension, or balance impairment (12, 13). The presence of multiple conditions has been linked to an increased risk of falls (10), and this might be associated with increased medication use or polypharmacy. A recent cross-sectional study among community-dwelling adults aged ≥50 years living in Saudi Arabia reported that the prevalence of falls was 12.6% and the risk of falls was significantly associated with the number of medications taken (OR 1.55, 95% CI [1.16, 2.07], *p* = 0.003) (14). Although the cross-sectional design of the study limits the ability to determine future fall incidence, the authors recommended that future longitudinal studies should examine the relationship between fall incidence, medications, and polypharmacy (14).

Polypharmacy has been defined as the use of four or more medications at the same time (15, 16). Previous research indicated that polypharmacy was associated with an increased risk of falls globally and in Saudi Arabia (17–21). However, previous research was limited by its cross-sectional design and focus on older adults (>60 years), particularly in Saudi Arabia. The Saudi population faces unique demographic and health challenges due to rapid population aging and the increased prevalence of chronic conditions (3–10), which often require multiple medications. The lack of research examining the association between the number of medications and/or polypharmacy and longitudinal fall incidence in Saudi Arabia presents challenges for healthcare management and fall prevention for this population. Limited longitudinal research from Western countries has investigated the association of the number of medications and polypharmacy with the risk of falls (22–25). These studies found that an increased number of medications and polypharmacy were associated with an increased risk of falls. However, definitions for polypharmacy and methods for evaluating fall risk differed across studies, with some defining polypharmacy as the use of five or more medications and using recall periods of 1–2 years to assess fall incidence (22–25).

The lack of prospective longitudinal studies may influence healthcare practices and fall prevention programs, as fall predictions might be limited to 1-year recall data. A critical gap exists in region-specific research, as the majority of studies on this topic originate from Western populations, which may not fully account for the genetic, lifestyle, and healthcare system differences in Saudi Arabia. A previous cross-sectional study from Saudi Arabia established the association of the number of medications and polypharmacy with a history of falls among participants aged 50 years and older (14). Thus, a follow-up study was conducted to examine fall incidence and medication use at two additional time points for the same participants. Therefore, this study aimed to explore the association of the baseline number of medications and polypharmacy with fall incidence at 4–6 months and 9-10 months of follow–up, among Saudi adults aged 50 years and older from the community.

## 2 Methods

### 2.1 Design and participants

This study was a prospective cohort study with up to 10 months of follow-up to assess fall incidence. All participants (*n* = 211) were recruited from community settings across multiple regions in Saudi Arabia, including Riyadh, Al-Kharj, Jazan, Arar, Hail, Jeddah, and Tabuk. Baseline data, including demographics, medications, and fall history, were collected between February and April 2022; follow-up data on fall incidence were collected at 4–6 months in August–September 2022, and final follow-up data on fall incidence were collected at 9-10 months in November 2022–January 2023. Participants were recruited through convenience sampling from community settings that included hospitals, clinics, malls, mosques, and other places. The inclusion criteria were as follows: (1) Saudi adults aged 50 years or older; (2) they must be able to read and write in Arabic to complete the self-reported measures; and (3) they must be able to ambulate independently. The exclusion criteria were as follows: the inability to read or write in Arabic and the inability to ambulate or perform functional tests safely. Each participant signed a consent form before enrollment and had the choice to withdraw from this study at any time. This study was approved by the Research Ethics Committee at Prince Sattam Bin Abdulaziz University (No. RHPT/021/017).

### 2.2 Clinical variables

#### 2.2.1 Fall incidence

Fall incidence was set as the main outcome, categorized into two groups: fallers and non-fallers. This was assessed at two time points: 4–6 months of follow-up (time 2) and 9-10 months of follow-up (time 3), in addition to baseline fall history (time 1). For feasibility, a time window was used during recruitment, and it was calculated in days as time to event (fall). Fall was defined as “inadvertently coming to rest on the ground” (26). At baseline, all participants were asked during the interview whether they had experienced a fall in the past 12 months. If the answer was yes, the participants were classified as fallers at baseline. For the follow-up periods, including time 2 and time 3, the majority of the participants were asked about fall incidence either by telephone or during a short interview when available. The question asked was, “Have you experienced a fall during the preceding 4–6 months or since the last visit?” Participants who answered yes were classified as fallers at follow-up times 2 and 3.

#### 2.2.2 Medications

The baseline number of medications (continuous variable) and baseline polypharmacy (categorical variable) were the exposure variables in the current study. Currently used medications were recorded and verified by participants. These medications included antidiabetics, antihypertensives, antilipemics, antiplatelets or anticoagulants, gastrointestinal medications, thyroid medications, pain medications, and antidepressants, among others. The number of currently used medications was recorded, calculated as the sum of all medications in use, excluding vitamin supplements. Polypharmacy was categorized based on the current use of four or more medications in the current study, as previous research on falls used this cutoff for polypharmacy (15–17, 27).

#### 2.2.3 Covariates

In addition to age, clinical variables that have been associated with fall incidence were included. Based on previous evidence and to limit the number of covariates to a minimal level due to the small number of fall events in the current study, baseline fall history (28), depressive symptoms (29), and insomnia score (30–32) were included as covariates. Depressive symptoms were assessed using the Arabic Patient Health Questionnaire-9 (PHQ-9) (33). The PHQ-9 is a validated and reliable questionnaire to assess depressive symptoms in primary care and other clinical settings, with higher scores indicating more depressive symptoms. The Arabic version of the Insomnia Severity Index (ISI), a validated and reliable 7-item screening tool, was used in this study to assess insomnia severity. It utilizes a 5-point Likert scale (0–4) for each item, where higher total scores indicate more severe insomnia symptoms (34).

### 2.3 Statistical analysis

Data were presented as means and counts to summarize the baseline demographics and clinical variables. Fall incidence was expressed as fall events per 100 person-years of observation. This measure was calculated as (total fall events/total person-years of follow-up) × 100, where person-years were derived by converting each participant’s actual assessment dates, calculating time to first fall (or censoring, whichever occurred first) in days, and converting to years. Participants who discontinued follow-up prior to the final assessment (9-10 months) but had completed the second assessment (4–6 months) were retained in survival analyses and right-censored at their last completed visit date, rather than excluded, consistent with standard practices for handling incomplete follow-up in time-to-event analysis. This resulted in an analytic sample of *n* = 205 for survival models.

To examine the longitudinal outcome data for fall incidence over up to 10 months of follow-up and the time-to-fall structure, survival analyses, including Cox proportional hazards regression with adjustment and backward likelihood-ratio selection, were utilized to estimate hazard ratios (HRs) and 95% confidence intervals (CIs) for the baseline number of medications and baseline polypharmacy in relation to the occurrence of a first fall event at times 2 and 3. Since fall events were ascertained only at discrete follow-up visits (baseline, 4–6 months, and 9-10 months), exact fall dates were unknown; each reported fall was therefore assigned the date of the visit at which it was first identified, and time-to-event was calculated in days from baseline to this assigned date. Participants who did not report a fall by their last completed visit were treated as censored at that visit date. This approach approximates continuous event timing from interval-censored, visit-based data and was selected for consistency with prior fall-risk research. The primary analysis used a standard Cox proportional hazards regression with backward likelihood ratio selection to retain covariates associated with time to first fall in the current study. A Kaplan–Meier curve was created to display fall incidence by polypharmacy status at times 2 and 3. The independent variables of interest, baseline number of medications and baseline polypharmacy, were included in the model one at a time as predictor variables to avoid multicollinearity between medication variables. Baseline fall was included as a covariate to improve the survival function because the model was created to examine the association of future fall incidence with baseline medications and polypharmacy. The models were adjusted for potential covariates that might be associated with falls, including age (as a continuous variable), baseline fall history (as a categorical binary variable with 0 and 1 codes), depressive symptoms using the PHQ-9 (as a continuous variable), and insomnia using the ISI score (as a continuous variable). This approach aimed to minimize the number of covariates due to the small number of events in the current study. The proportional hazards assumption was tested using Schoenfeld residuals. Schoenfeld residuals were not statistically significant (*p* = 0.91), indicating that there was no violation of the proportional hazards assumption. Missing data were treated as listwise deletion.

As a sensitivity analysis, we used Firth-penalized Cox regression adjusted for the candidate covariates, including age, baseline fall history, depressive symptoms using the PHQ-9, and insomnia using the ISI score. Firth-penalized Cox regression was used to address the small-sample bias and the limited number of events in the current study. Because exact fall dates were unknown and events were ascertained only at discrete follow-up visits, we conducted a parametric interval-censored Cox proportional hazards model (icenReg package, R, Weibull baseline hazard) using the true lower and upper bounds of the interval in which each fall could have occurred, derived from participants’ actual assessment dates, rather than assigning falls to a single visit date. This model used the same covariate set as the primary and Firth-penalized Cox analyses. Analyses were conducted using IBM SPSS Statistics for Mac, version 25.0 (IBM Corp., Armonk, NY), and R version 4.6.1. The alpha level was set at 0.05.

## 3 Results

### 3.1 Participant characteristics

A total of 211 participants were included in this study with a mean follow-up duration of 8.5 months. The median follow-up time was 274 days, ranging from 117 days to 329 days of follow-up. The mean age of the participants was 59.48 ± 7. The mean PHQ-9 score was 5.11 ± 5 among the sample. The mean ISI score was 10.12 ± 6.22, and the mean number of medications used was 0.95 ± 1.3, with 6.6% of participants classified as having polypharmacy. Baseline variables are shown in Table 1. No participant with polypharmacy was lost to follow-up (0/14), and 12 of 197 non-polypharmacy participants (6.1%) dropped out. Fisher’s exact test confirmed that this difference was not statistically significant (*p* = 0.43), indicating completion status did not differ by baseline polypharmacy status. The reasons for attrition were lack of interest in the study and no response from participants.

**Table 1:** Baseline demographics and clinical characteristics (*n* = 211).

Variables	Mean ± SD or *n* (%)
Age, years	59.48 ± 7
Sex (female/male)	112 (53)/99 (47)
PHQ-9 score	5.05 ± 5.11
ISI score	10.12 ± 6.22
Number of medications, mean ± SD	0.95 ± 1.3
Polypharmacy	
≥4 medications	14 (6.6)
<4 medications	197 (93.4)
Baseline fall history (*n* = 211)	26 (12.3%)
Fall incidence at 4–6 months of follow-up (*n* = 205)	18 (8.8%)
Fall incidence at 9–10 months of follow-up (*n* = 199)	28 (14.1%)

SD, standard deviation; PHQ-9, Patient Health Questionnaire-9; ISI, Insomnia Severity Index.

### 3.2 Fall incidence

A total of 55 (26.1%) participants experienced at least one fall from baseline through 10 months of follow-up. A total of 37 (17.5%) participants experienced at least one fall during the follow-up period at 4–6 months and 9-10 months of follow-up. The overall fall incidence rate was 24.84 per 100 person-years. A Kaplan–Meier curve was generated and is shown in Figure 1. At baseline, fall history was 12.3%. The percentage of fall incidence at the 4–6-month follow-up was 8.8%. The percentage of fall incidence at the 9–10-month follow-up was 14.1%, as shown in Table 1. These falls from baseline to the end of follow-up (*n* = 72 falls) included recurrent falls, as some participants fell at baseline and during follow-up, thereby increasing the fall events beyond unique falls (*n* = 55 falls). Fall incidence was 31 falls (15.7%) in the unexposed group without polypharmacy compared with 6 falls (42.9%) in the exposed group with polypharmacy. Person-years estimates based on group-specific rates were 22.26 per 100 person-years in the non-polypharmacy group and 62.24 per 100 person-years in the polypharmacy group, as shown in Table 2.

**Figure 1:**
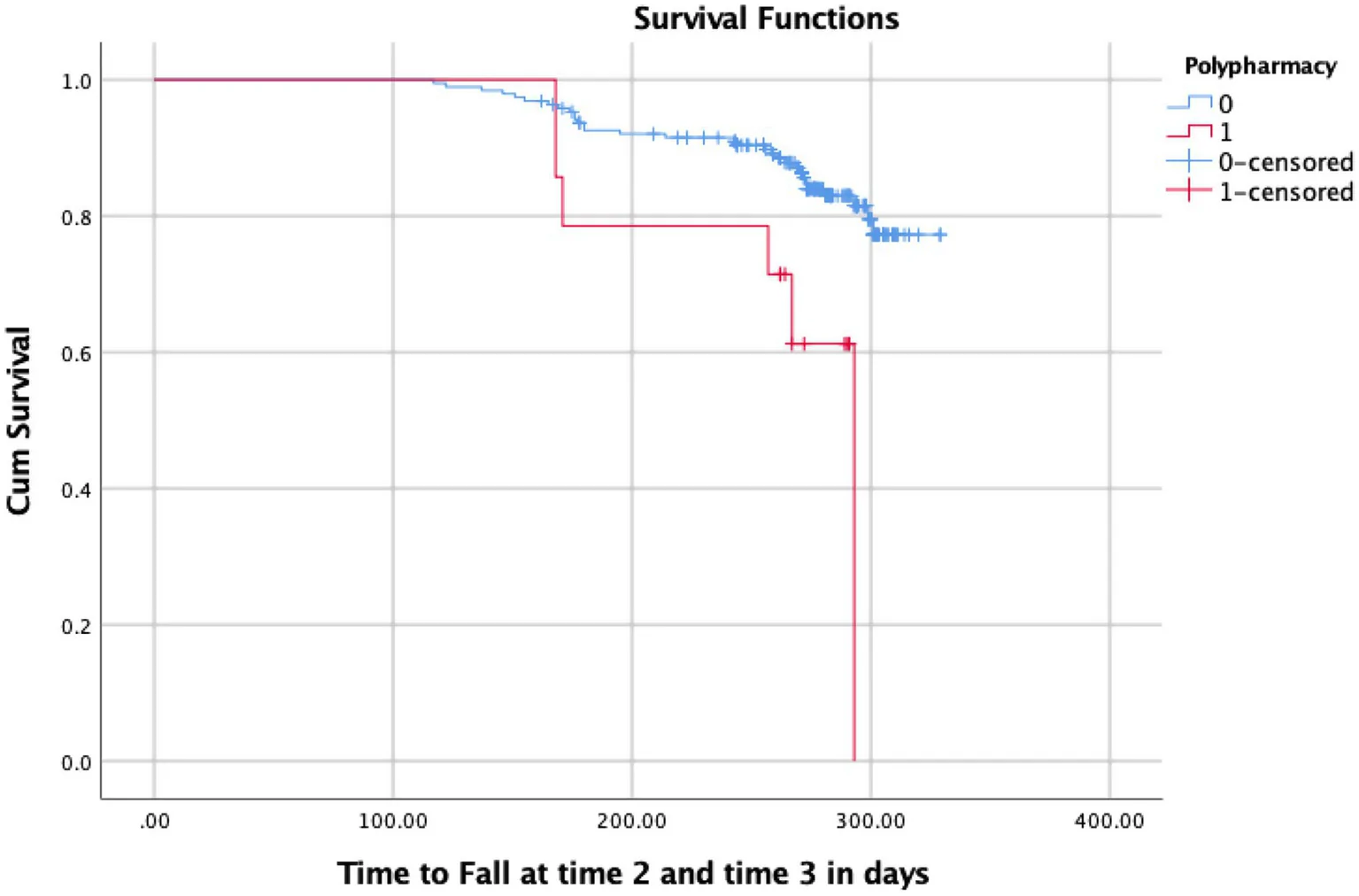
Kaplan–Meier curve for polypharmacy and fall incidence at 4–6 months and 9-10 months of follow-up.

**Table 2:** Fall incidence table at time 2 (4–6 months of follow-up) and time 3 (9–10 months of follow-up).

Groups	Events	Person-years	*N*	Rate per 100 person-years
Non-polypharmacy	31	139.29	197	22.26
Polypharmacy	6	9.64	14	62.24
Overall	37	148.93	211	24.84

### 3.3 Association between medications and fall risk

Cox proportional hazards regression with backward likelihood ratio was used to investigate whether baseline number of medications was associated with fall incidence over time at 4–6 and 9-10 months of follow-up, as shown in Table 3. The unadjusted model (*n* = 205) showed that the baseline number of medications was significantly associated with fall incidence (HR: 1.32, 95% CI: 1.08, 1.62; *p* = 0.007). However, the number of medications was not significantly associated with fall incidence (HR: 1.23, 95% CI: 0.97, 1.54; *p* = 0.08) in the final parsimonious model (*n* = 205) retaining age and ISI score. The results of the sensitivity analysis are shown in Table 4. The Firth-penalized Cox model (n = 205) showed that there was no significant association between baseline number of medications and fall incidence (HR: 1.15, 95% CI: 0.88, 1.44; *p* = 0.28) after adjustment for covariates including age, baseline fall history, depressive symptoms using PHQ-9 score, and insomnia measured using the ISI score. Finally, the results of the interval-censored Weibull analysis (*n* = 205) showed no significant association between baseline number of medications and fall incidence (HR: 1.11, 95% CI: 0.87, 1.42; *p* = 0.41) after adjustment for covariates.

**Table 3:** Cox proportional hazards regression for the associations of the number of medications and polypharmacy with fall events.

Variables	HR (95% CI)	*p*-value
Number of medications (unadjusted model)	1.32 (1.08, 1.62)	0.007
Number of medications (final adjusted model)	1.23 (0.97, 1.54)	0.08
Age (final adjusted model)	1.05 (1.01, 1.09)	0.020
ISI score (final adjusted model)	1.09 (1.04, 1.15)	0.001
Polypharmacy (unadjusted model)	3.73 (1.54, 9.06)	0.012
Polypharmacy (final adjusted model)	3.99 (1.63, 9.81)	0.003
Age (final adjusted model)	1.05 (1.02, 1.09)	0.006
ISI score (final adjusted model)	1.11 (1.05, 1.16)	<0.001

Adjusted models were adjusted for potential variables including age, baseline fall, depressive symptoms measured using PHQ-9, and insomnia measured using the ISI score with backward likelihood-ratio selection. The polypharmacy and number-of-medications models used Cox regression with backward likelihood-ratio selection; the final parsimonious model retained age and ISI score. HR, hazard ratio; CI, confidence interval; ISI, Insomnia Severity Index; PHQ-9, Patient Health Questionnaire-9.

**Table 4:** Sensitivity analysis using Firth-penalized Cox and Interval-censored Weibull analyses for number of medications and polypharmacy with fall events.

Sensitivity analysis	Variables	HR (95% CI)	*p*-value
Firth-penalized Cox	Number of medications	1.15 (0.88, 1.44)	0.28
	Polypharmacy	3.09 (1.10, 7.64)	0.034
Interval-censored Weibull	Number of medications	1.11 (0.87, 1.42)	0.41
	Polypharmacy	2.79 (1.02, 7.62)	0.046

Models were adjusted for age, baseline fall, depressive symptoms measured using PHQ-9, and insomnia measured using the ISI score. HR, hazard ratio; CI, confidence interval; PHQ-9, Patient Health Questionnaire-9; ISI, Insomnia Severity Index.

### 3.4 Association between polypharmacy and fall risk

Cox proportional hazards regression was used to investigate whether baseline polypharmacy was associated with fall incidence over time at 4–6 and 9-10 months of follow-up, as shown in Table 3. The unadjusted model (*n* = 205) showed that polypharmacy was associated with an approximately 3.7-fold increase in risk of fall incidence (HR: 3.73, 95% CI: 1.54, 9.06; *p* = 0.012). Using a Cox proportional hazards regression model (*n* = 205) with backward likelihood-ratio selection, polypharmacy was associated with an approximately fourfold increase in risk of fall incidence (HR: 3.99, 95% CI: 1.63, 9.81; *p* = 0.003) in the final parsimonious model, which retained age and insomnia measured using the ISI score as significant covariates. The results of the sensitivity analysis are shown in Table 4. Firth-penalized Cox regression analysis (*n* = 205) showed that there was a significant association between baseline polypharmacy and fall incidence (HR: 3.09, 95% CI: 1.10, 7.64; *p* = 0.034) after adjustment for covariates including age, baseline fall history, depressive symptoms using the PHQ-9 score, and insomnia measured using the ISI score. Finally, the results of the interval-censored Weibull analysis (*n* = 205) showed a significant association between baseline polypharmacy and fall incidence (HR: 2.79, 95% CI: 1.02, 7.62; *p* = 0.046) after adjustment for covariates.

## 4 Discussion

This study investigated the association of baseline medication counts and polypharmacy with longitudinal fall incidence over up to 10 months of follow-up among community-dwelling Saudi adults aged 50 years and older. The findings indicated a significant association between polypharmacy and fall incidence during follow-up. The number of medications at baseline was not associated with future fall incidence in the current study after adjustment for potential covariates. This study was the first longitudinal study examining the association between baseline medication use and fall incidence over a period of up to 10 months in Saudi Arabia.

The current finding of no association between the baseline number of medications and fall incidence was inconsistent with previous research using longitudinal cohort designs (22, 23). The unadjusted model showed a significant association between baseline number of medications and fall incidence over time. However, after adjustment for potential covariates, this association was not significant, indicating a confounding effect of the covariates on this association. Previous studies found that an increased number of medications was associated with increased fall incidence (22, 23). However, these studies examined falls using different approaches and follow-up periods among adults aged 65 years and older, who were older than the current study (≥50 years) (22, 23). Ie et al. (23) examined fall incidence using monthly phone calls over 12 months. The number of medications and their classifications in the study by Ie et al. (23) were extracted from prescription claims. However, the current use of these medications may differ because dispensing records do not reflect the current use of medications. The other study by Yoshida et al. (22) included participants aged >69 years, a population relatively older than the current study age group. Although this study involved a 3-year follow-up, the assessment of falls was limited to the past 12 months at the third year (22). Close monitoring for fall incidence is crucial, particularly for older persons. The current study examined fall risk at baseline, 4–6 months of follow-up, and 9-10 months of follow-up. This approach may allow close monitoring of participants and minimize recall bias over 12 months or more. The finding of no association between number of medications and future fall incidence might be explained by the low fall incidence and adjustment for possible covariates that might be associated with future fall incidence, such as baseline fall history, depressive symptoms score, and other covariates. Future research should examine the classes of medications and the association of changes in medications over time with future fall incidence to comprehensively identify the relationship between medications and fall incidence.

Future fall incidence was associated with baseline polypharmacy in the current study. The findings from the current work were consistent with past reports (24, 25). Richardson et al. (25) found an association between polypharmacy and the risk of any fall and the number of falls among community-dwelling adults aged 50 years and older. However, this study differed from the current study. For example, fall incidence was evaluated 2 years after the baseline examination for polypharmacy. The definition of polypharmacy (≥5 medications) differed from the current study (≥4 medications) (25). Polypharmacy may be defined as the use of four or more medications for younger age groups, according to the inclusion criteria used to identify those at risk. Another study from England included participants aged 60 years and older during wave 6 of follow-up visits (24). Their findings were consistent with the current study regarding the significant association between baseline polypharmacy and increased risk of fall incidence (24). Although this study used multiple cutoffs for polypharmacy, including four or more medications, which was consistent with the current study, the evaluation of fall incidence was over the preceding 2 years, in contrast with the current study (4–6-month window). Therefore, future research should examine fall incidence with close monitoring within a 1-year window to minimize participant recall bias.

The association between fall incidence and polypharmacy might be explained by several mechanisms. First, the cumulative use of multiple medications may lead to complex drug interactions and thereby increase the risk of falls. Physiologically, the consumption of certain medication classes is associated with adverse events that affect neuromuscular, digestive, and cognitive functions (17). A meta-analysis of 281 studies that examined the associations between fall risk and different types of medications found that both opioid and antiepileptic use were significantly associated (OR = 1.60 and 1.55, respectively) with an increased risk of falls (17). Opioids may contribute to falls by causing sedation, dizziness, and cognitive impairment (17). Antiepileptics are also associated with dizziness, unsteadiness, and blurred vision, which impair physical stability and may lead to fall incidence. However, the current study did not examine medication classifications, and future research should examine this association comprehensively.

The strengths of this study were primarily the prospective longitudinal design of the study with close monitoring of fall incidence among participants at baseline and two additional time points. In addition, the current study was the first study conducted in Saudi Arabia to examine medications and fall incidence using a longitudinal design. However, this study has some limitations that should be considered in future studies. The sampling technique used a convenience sampling approach that may limit the generalizability of the findings because participants were not randomly selected. Although the design was longitudinal with 9 months of follow-up, this duration is short and may limit the generalizability of the current findings. Fall history at baseline and fall incidence during follow-up might be affected by participant recall bias. During follow-up, fall incidence was obtained from some of the participants by telephone, which might affect the results of the current study. Furthermore, the current study is potentially vulnerable to residual confounding because certain factors, such as changes in health status, functional decline, and environmental hazards, could have changed over the 10-month follow-up period; these were not properly addressed and might affect the risk of falls. Additionally, data on the types of medications were not collected, thereby limiting the analysis of the role of medication class in fall incidence. Future research should investigate fall-risk-increasing drugs in this population. In the current study, baseline polypharmacy was used without capturing changes in medications over time. Therefore, there is a risk of time-varying confounding that a baseline-fixed exposure cannot capture, and this should be acknowledged and considered in future research. A key limitation of the current study is that medication use was ascertained through participant self-report during a structured interview rather than through verification against prescription or pharmacy records. The mean number of medications reported at baseline (0.95 ± 1.3) is lower than would typically be expected in a population of this age with a comparably high prevalence of multimorbidity (45%), suggesting that self-report may have led to under-ascertainment of true medication use, particularly for over-the-counter and as-needed medications. This exposure misclassification may have attenuated or otherwise distorted the observed associations between medication counts, polypharmacy, and fall incidence, and should be considered when interpreting the study’s findings. Verification against pharmacy or prescription records was not feasible for this community-recruited sample. Future research should prioritize objective medication verification where feasible. The sample size is relatively small, and future research should conduct a power calculation to estimate the appropriate sample size. Fall events were ascertained at discrete assessment visits rather than continuously, making the underlying data interval-censored; assigning falls to the reporting visit date is a common approximation in the literature but does not fully capture the true timing uncertainty. Interval-censored or discrete-time survival models represent a more methodologically precise alternative for future research using this assessment structure, and the results of the interval-censored analysis in the current study were consistent with the main Cox proportional hazards analysis. The mean number of medications at baseline was relatively low for a population aged 50 years and older. This raises the possibility of under-ascertainment of self-reported medications and resulting exposure misclassification that may affect the results. Although the models were adjusted for potential covariates, the events-per-variable ratio was relatively low, which may raise overfitting concerns.

## 5 Conclusion

This exploratory study suggests a possible association between baseline polypharmacy and fall incidence over up to 10 months of follow-up among community-dwelling Saudi adults aged 50 years and older. However, these findings were based on a small polypharmacy subgroup (*n* = 14) with only 6 events and should be interpreted with caution. These findings may support further investigation of medication and polypharmacy screening as part of broader fall-risk management strategies in community-dwelling older adults, although further research is needed to confirm this association and clarify its clinical implications.

## Data Availability

The raw data supporting the conclusions of this article will be made available by the authors, without undue reservation.
